# Conservative Management of Extensive Type V Aplasia Cutis Congenita in a Term Neonate Following Fetus Papyraceus: A Case Report and Guide for Outpatient Pediatric Management

**DOI:** 10.1155/crpe/4750310

**Published:** 2026-08-23

**Authors:** Kennedy Sabharwal, John Meadows

**Affiliations:** ^1^ Department of Internal Medicine, The University of Tennessee Health Science Center, Knoxville, Tennessee, USA, tennessee.edu; ^2^ Department of Dermatology, Medical College of Georgia at Augusta University, Augusta, Georgia, USA, augusta.edu; ^3^ Department of Obstetrics and Gynecology, The University of Tennessee Health Science Center, Knoxville, Tennessee, USA, tennessee.edu

**Keywords:** aplasia cutis congenita, case report, conservative wound management, fetus papyraceus, monochorionic twin pregnancy

## Abstract

**Background:**

Type V aplasia cutis congenita (ACC) associated with fetus papyraceus presents management challenges for general pediatricians. While surgical intervention is often considered for extensive defects, conservative management may be effective with appropriate patient selection and protocols. To our knowledge, this is one of the most extensive cases of Type V ACC managed conservatively in the outpatient literature, and it provides a reproducible wound care protocol where none currently exists.

**Case:**

We report a female infant born at 37 weeks gestation with extensive Type V ACC involving circumferential areas of the torso, hips, posterior thighs, and posterior scalp (∼37–40% BSA) following intrauterine demise of a monochorionic co‐twin. Conservative management using moist wound healing principles with Aquaphor, nonadherent dressings, and multidisciplinary care resulted in complete re‐epithelialization within 6 weeks without surgical intervention. Outpatient management was successfully implemented with comprehensive family education and home health support.

**Conclusion:**

Even extensive, circumferential Type V ACC can be managed successfully without surgery in hemodynamically stable infants who receive structured conservative wound care and family education. General pediatricians should consider this approach as a reasonable first‐line strategy before surgical referral, reserving surgery for cases with failed conservative management, infection unresponsive to therapy, or significant functional impairment.

## 1. Introduction

Aplasia cutis congenita (ACC) is a rare congenital absence of skin present at birth, with an estimated incidence of 1 in 10,000 live births. Type V ACC, associated with fetus papyraceus in monochorionic twin pregnancies, represents a distinct clinical entity characterized by symmetric, often extensive skin defects resulting from vascular disruption following co‐twin demise. While large circumferential defects traditionally prompt surgical consultation, accumulating evidence supports conservative management as a viable first‐line approach for hemodynamically stable infants.

General pediatricians may encounter Type V ACC in their practice but often lack guidance on outpatient management strategies. To our knowledge, this represents one of the most extensive cases of Type V ACC successfully managed conservatively in the outpatient setting, providing a reproducible protocol where none currently exists. This case report demonstrates successful conservative management of extensive circumferential Type V ACC and provides practical protocols for pediatricians to implement evidence‐based wound care in the outpatient setting, potentially avoiding surgical intervention while achieving excellent outcomes.

## 2. Case Presentation

A female infant was born at 37 weeks of gestation via spontaneous vaginal delivery following a monochorionic monoamniotic twin pregnancy complicated by the intrauterine demise of the co‐twin at 12 weeks of gestation. The mother was a 41‐year‐old G6P4 with chronic hypertension and gestational diabetes mellitus. Maternal prenatal testing showed GBS positive status (treated with adequate intrapartum antibiotic prophylaxis), negative hepatitis B and C, negative HIV, nonreactive RPR, rubella immunity, and negative gonorrhea and chlamydia. Medications during pregnancy included prenatal vitamins and nifedipine for hypertension. Family history was unremarkable for genetic or congenital anomalies. Birth weight was 2920 g with Apgar scores of 8 and 9 at 1 and 5 min, respectively.

**FIGURE 1:**
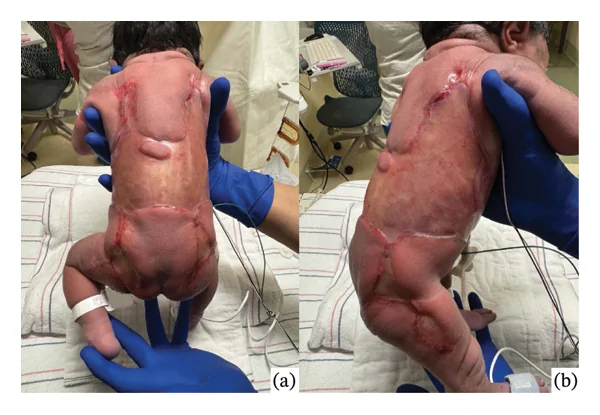
(a and b) Extensive circumferential Type V aplasia cutis congenita at birth involving torso, bilateral hips, posterior thighs, and posterior scalp following fetus papyraceus in a 37 week infant.

**FIGURE 2:**
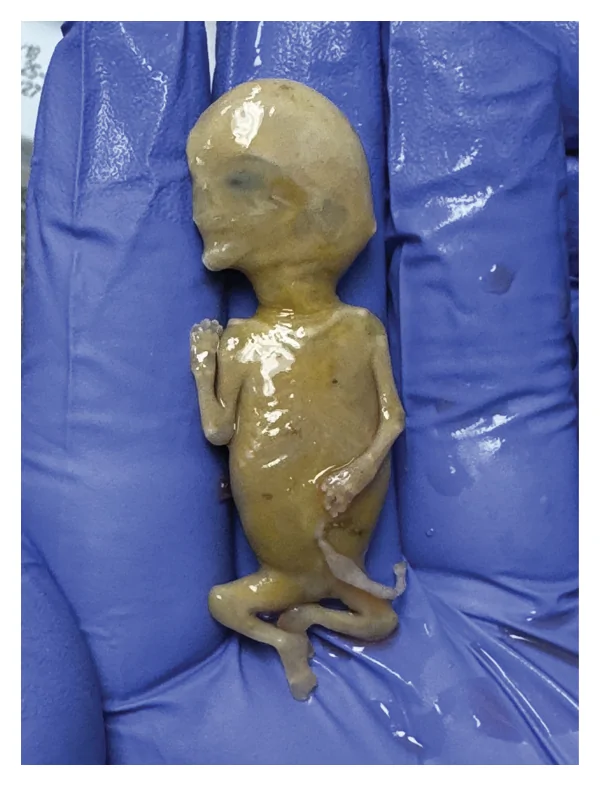
Mummified co‐twin (fetus papyraceus) whose intrauterine demise at 12 weeks gestation resulted in Type V aplasia cutis congenita in the surviving twin through vascular disruption mechanisms.

At birth, the infant was noted to have large, circumferential areas of absent skin involving most of the torso circumferentially, bilateral posterior scalp, bilateral hips, and abdomen wrapping around to the back (∼36–40% body surface area [BSA]). These defects appeared as well‐demarcated areas with complete absence of epidermis and dermis, exposing underlying subcutaneous tissue. (Figure 1a, b) The lesions were consistent with Type V ACC in the setting of fetus papyraceus. (Table 1) No other congenital anomalies were identified on physical examination. Of note, the demise twin was subsequently delivered as a fetal papyraceus, as seen in Figure 2.

**TABLE 1:** Frieden classification of aplasia cutis congenita.

Type	Name/description	Location of defect	Anomalies	Inheritance pattern	Management	Prognosis
1	Scalp ACC without multiple anomalies	Scalp vertex near hair whorl	None	Autosomal dominant or sporadic	Conservative wound care; surgery if large or skull defect	Excellent for small lesions; heals with alopecia scar
2	Scalp ACC with limb anomalies (Adams‐Oliver syndrome)	Midline scalp	Distal limb reduction defects	Autosomal dominant or recessive (NOTCH1, DLL4, ARHGAP31)	Multidisciplinary; wound care + ortho/genetics	Depends on limb severity; scalp lesions heal well
3	Scalp ACC with epidermal/sebaceous nevi	Scalp	Organoid or sebaceous nevi (mosaic RASopathy)	Sporadic mosaic mutation	Wound care; dermatology surveillance of nevi	Good for skin lesion; monitor nevi
4	ACC overlying embryologic malformations	Midline scalp or trunk	Neural tube defects, encephalocele, meningocele; hair collar sign	Sporadic	Urgent neurosurgical evaluation; surgical repair	Depends on central nervous system anomaly; can be severe
**5**	**ACC with fetus papyraceus**	**Trunk, flanks, hips,** **proximal limbs; scalp rare**	**Co-twin demise; single umbilical artery**	**Not inherited (vascular disruption)**	**Conservative wound care; surgery rarely needed**	**Favorable; heals with scarring**
6	ACC with epidermolysis bullosa (Bart syndrome)	Lower extremities common	EB blistering,nail dystrophy	Autosomal dominant	EB‐specific wound care; avoid trauma	Variable; depends on EB severity
7	ACC of extremities without EB	Extremities	Limb defects without EB	Variable/sporadic	Conservative care; orthopedic surgery consult	Good; usually heals well
8	ACC due to teratogens or intrauterine infection	Variable	Varicella, HSV, or drug exposure (methimazole,valproate, etc.)	Not inherited	Wound care; infection workup; medication review	Generally good if isolated
9	ACC with malformation syndromes	Variable, often scalp	Trisomy 13, Wolf‐Hirschhorn, Setleis, Johanson‐Blizzard, etc.	Chromosomal/syndromic	Genetics referral; individualized care	Driven by underlying syndrome

*Note:* The nine subtypes of ACC are categorized by lesion location, associated anomalies, inheritance pattern, management, and prognosis. Type V (bold), the focus of this report, is characterized by vascular disruption following co‐twin demise and most commonly presents with truncal and proximal extremity involvement [1–4].

Abbreviations: ACC, aplasia cutis congenita; EB, epidermolysis bullosa; HSV, herpes simplex virus.

Initial neonatal workup was performed to evaluate for underlying conditions and associated anomalies. Laboratory studies included complete blood count (admission hematocrit 42.8% and discharge hematocrit 35%), comprehensive metabolic panel, and infectious screening labs, all of which were reassuring. Blood culture was negative. Additional infectious workup beyond routine cultures was performed and remained reassuring. Genetic testing with nonrapid whole genome sequencing was performed and was normal. The differential diagnosis included genetic syndromes associated with aplasia cutis, such as Adams–Oliver syndrome and focal dermal hypoplasia, which were ruled out by normal genetic testing and clinical presentation.

Cranial ultrasound on hospital Day 1 demonstrated normal anatomy with no intraventricular hemorrhage, but it could not exclude Grade 1 periventricular leukomalacia. Repeat imaging on Day 8 showed transient left Grade 1 germinal matrix hemorrhage (resolved by Day 18) with no periventricular leukomalacia. Echocardiography showed small atrial septal defect (ASD) and small patent ductus arteriosus (PDA) with left‐to‐right shunting; follow‐up echo showed patent foramen ovale (PFO) versus ASD with bilateral physiologic peripheral pulmonic stenosis. Ophthalmologic evaluation revealed scattered retinal hemorrhages consistent with vaginal delivery, which improved on follow‐up examination.

The infant was admitted to the neonatal intensive care unit for monitoring and evaluation. A multidisciplinary team consisting of neonatology, dermatology, plastic surgery, and wound care specialists recommended a conservative treatment approach. The wound care protocol included gentle cleansing, application of Aquaphor, nonadherent Telfa dressings, and Kerlix gauze wrapping with dressing changes initially every 8 h, increased to every 6 h for posterior thigh areas due to dressing displacement from movement. Silver sulfadiazine was avoided due to concerns about systemic absorption given the extensive surface area involvement and absence of infection.

Pain management during dressing changes was provided with acetaminophen every 6 h as needed and intranasal fentanyl during hospitalization only under continuous monitoring. Intranasal fentanyl was discontinued prior to discharge as pain tolerance improved with epithelialization, and only acetaminophen was continued for outpatient management. The infant received empiric antibiotics (ampicillin and gentamicin) for 48 h with negative blood cultures. Standard neonatal feeding was initiated with breast milk and advanced as tolerated.

**FIGURE 3:**
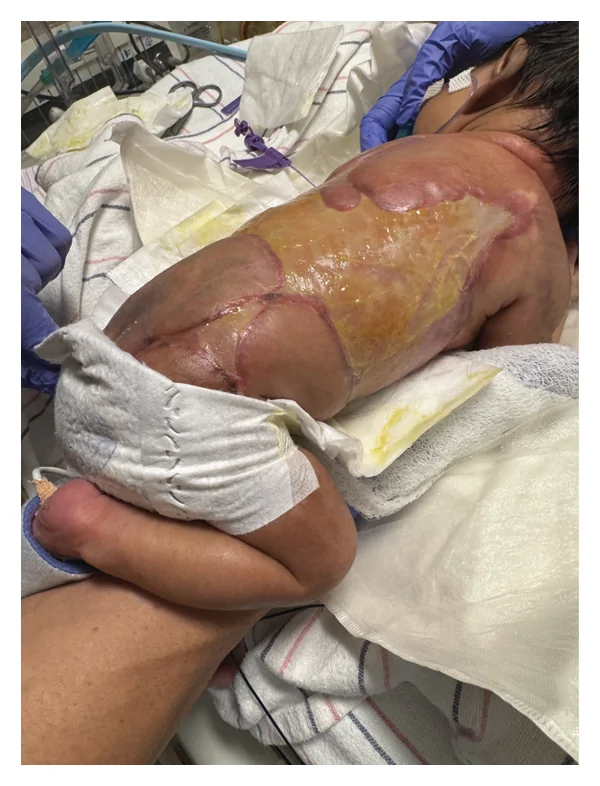
Hospital Day 5 of conservative management showing early granulation tissue formation and progressive wound healing with moist wound care using Aquaphor and Telfa dressings.

**FIGURE 4:**
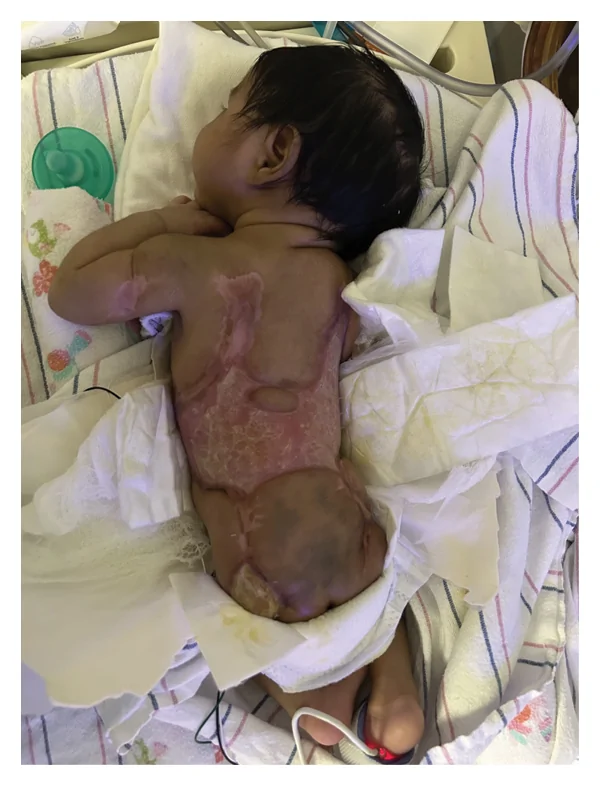
Hospital Day 20 (day of discharge) showing significant re‐epithelialization of all defects following 3 weeks of conservative management without surgical intervention.

Throughout the NICU course, the infant remained hemodynamically stable and tolerated feeds well. Progressive wound healing was observed beginning on hospital Day 3, with granulation tissue formation noted at the wound edges (Figure 3). Pain and discomfort during dressing changes decreased significantly by the second week as epithelialization progressed. During her 20 day NICU admission, the wounds demonstrated granulation and re‐epithelialization without surgical intervention (Figure 4). At the time of discharge, many of the defects showed pink granulation tissue with moist, epithelializing areas. Discharge weight was 3180 g (birth weight 2920 g). The infant was discharged home with a wound care regimen using Aquaphor and Telfa dressings every 8 h. No adverse events attributable to the conservative wound care protocol occurred during the treatment course.

**FIGURE 5:**
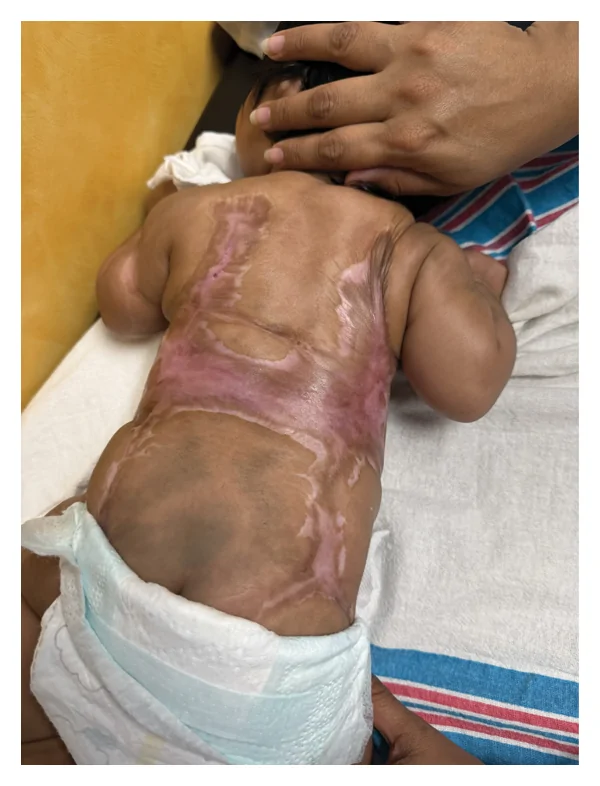
4 month well‐child visit showing mixed‐stage wound healing with peripheral epithelialization, central granulation tissue, hyperpigmented margins, and mild atrophic scarring.

At the 4‐month pediatric appointment (15 weeks postdischarge), the patient demonstrated progressive wound healing with complete epithelialization of peripheral zones, ongoing granulation of central areas, postinflammatory hyperpigmentation, mild scarring, and no evidence of infection or functionally limiting contractures (Figure 5, Table 2). No additional laboratory or imaging studies were obtained at follow‐up; assessment was based on clinical examination alone.

**TABLE 2:** Timeline of the patient’s clinical course from prenatal diagnosis through 4 month follow‐up.

Time	Event
12 weeks gestation	Intrauterine demise of monochorionic co‐twin (fetus papyraceus)
Birth (37 weeks gestation)	Spontaneous vaginal delivery; extensive circumferential Type V ACC noted (∼36–40% BSA); Apgar 8 and 9 at 1 and 5 min; birth weight 2920 g
Day 1	Admitted to NICU; cranial ultrasound normal, could not exclude Grade 1 periventricular leukomalacia; multidisciplinary team (neonatology, dermatology, plastic surgery, and wound care) recommends conservative management; empiric ampicillin/gentamicin started
Day 3	Progressive wound healing begins; granulation tissue noted at wound edges
Day 5	Continued granulation and wound healing on conservative protocol (Figure 3)
Day 8	Repeat cranial imaging shows transient left Grade 1 germinal matrix hemorrhage; no periventricular leukomalacia
Day 18	Germinal matrix hemorrhage resolved
Day 20	Discharged home; significant re‐epithelialization of all defects (Figure 4); discharge weight 3180 g; discharged on Aquaphor/Telfa dressing regimen every 8 h
15 weeks postdischarge (4 month visit)	Complete epithelialization of peripheral zones, ongoing central granulation, postinflammatory hyperpigmentation, mild scarring, no infection or functionally limiting contractures (Figure 5)

Written informed parental consent was obtained for publication of this case report and photographs.

## 3. Discussion

This case demonstrates how extensive Type V ACC can be successfully managed by general pediatricians using conservative outpatient wound care after initial hospital stabilization. While surgical management is often considered for large defects, our experience supports a conservative approach that can be safely implemented in the outpatient setting.

Type V ACC represents a distinct subtype associated with fetus papyraceus, thought to result from vascular disruption in utero due to thromboembolic or hypoperfusion events after the demise of a monochorionic co‐twin [5]. The pathophysiology involves arteriovenous vascular anastomoses present in 90%–95% of monochorionic placentas, which facilitate rapid blood shunting from the surviving fetus to the dying one during intrauterine demise [6, 7]. This acute fetal‐fetal transfusion induces acute hypovolemia in the living twin, resulting in ischemic necrosis of developing skin, especially in areas with vulnerable circulation like the trunk and extremities [8, 9].

There is currently no established consensus guideline for the management of Type V ACC, and treatment decisions are largely guided by case reports and expert opinion [10]. General pediatricians may consider managing extensive Type V ACC in the outpatient setting when specific criteria are met: hemodynamically stable with normal vital signs and adequate feeding, absence of underlying bone defects, no active infection, reliable family support with demonstrated wound care competency, and access to pediatric subspecialty consultation if needed [11]. Surgical management of large ACC defects can involve multiple operations over months to years, including skin grafting and tissue expansion (where a device is placed under nearby skin and slowly inflated to grow enough skin to cover the defect), all of which may be avoided with successful conservative management in stable patients [1]. Genetic evaluation should also be considered, as whole genome sequencing may be indicated to rule out associated syndromes.

General pediatricians should involve plastic surgery or dermatology for wounds showing no healing progress after 3‐4 weeks of conservative management, signs of infection not responding to appropriate antibiotic therapy, development of contractures affecting range of motion/function, concern for underlying bone involvement, or significant parental anxiety requiring additional reassurance and expertise [11, 12]. Emergency consultation is warranted for signs of sepsis, significant fluid imbalances, or rapid deterioration (Table 3).

**TABLE 3:** Conservative wound care protocol for Type V aplasia cutis congenita.

Parameter	Recommendation
Primary wound agent	Petrolatum‐based ointment (e.g., Aquaphor and Vaseline); avoid silver sulfadiazine in neonates due to risk of bilirubin displacement from albumin
Dressing type	Nonadherent primary layer (e.g., Telfa pad) and absorbent secondary wrap (e.g., Kerlix gauze)
Change frequency	Every 8–12 h initially; adjust based on wound drainage, dressing, and anatomic location (e.g., high‐mobility areas may require more frequent changes)
Wound cleansing	Gentle irrigation with normal saline or clean water at each change
Pain control (inpatient)	Acetaminophen scheduled or as needed; short‐term intranasal fentanyl for dressing changes under continuous monitoring in early healing phase
Pain control (outpatient)	Acetaminophen as needed; frequency reduced as epithelialization progresses and pain tolerance improves
Infection monitoring	Assess at each dressing change for erythema, purulent exudate, malodor, fever, or feeding changes; obtain wound or blood culture if infection suspected
Criteria for escalation	No epithelialization after 3‐4 weeks; wound enlargement; infection unresponsive to antibiotics; contracture with functional limitation; underlying dural involvement
Outpatient follow‐up	Weekly visits during initial 2‐3 weeks; wound photography and measurement at each visit to document progress
Expected healing timeline	Granulation tissue by days 3–5; significant re‐epithelialization by 3 weeks; complete healing by 6–8 weeks in uncomplicated cases

*Note:* Parameters are based on the management approach used in this case and existing literature. Recommendations should be adapted to the individual patient’s clinical status, wound extent, and care setting.

Successful outpatient management requires comprehensive family education, including proper wound care, recognition of complications, pain management strategies, and when to seek medical attention [13]. Coordination with medical supply companies for dressing materials and home health services for initial support can significantly improve outcomes. Physical and occupational therapy consultation should be arranged early to prevent contractures and optimize functional outcomes.

Strengths of this report include detailed wound care documentation and photographic follow‐up spanning the full course of epithelialization, providing pediatricians with reproducible, real‐world guidance for outpatient management. The success of conservative management in our case was attributed to several factors: the infant was term and clinically stable at birth with no evidence of infection, a consistent team approach was maintained, wound healing principles were emphasized with gentle handling and appropriate pain control, and comprehensive family education enabled transition to outpatient care. The avoidance of silver sulfadiazine minimized systemic toxicity while maintaining wound healing.

Limitations of this report include the qualitative nature of outpatient wound assessment, absence of standardized wound measurement tools, and a follow‐up period of 4 months, which limits conclusions about long‐term scarring, alopecia, or functional outcomes.

## 4. Conclusion

We recommend conservative management as the first‐line approach for stable neonates with Type V ACC. This can avoid surgical risks while achieving wonderful clinical outcomes. Surgical intervention should be reserved for cases with underlying cranial defects with dural exposure, failed conservative management after 4–6 weeks, signs of infection unresponsive to appropriate therapy, or significant functional impairment due to contracture formation. The most crucial elements include maintaining optimal wound moisture, preventing infection, providing adequate pain control, and ensuring family education for successful outpatient management.

## Funding

No funding was obtained for this work.

## Ethics Statement

IRB approval was not required for this manuscript. This is a single‐patient case report, which does not constitute human subjects research under 45 CFR 46. Written informed parental consent was obtained for publication of this case report and all clinical photographs.

## Conflicts of Interest

The authors declare no conflicts of interest.

## Data Availability

The data that support the findings of this study are available from the corresponding author upon reasonable request.
